# The Use of Assessment of Chronic Illness Care Technology to Evaluate the Institutional Capacity for HIV/AIDS Management

**DOI:** 10.3389/fphar.2019.00165

**Published:** 2019-02-27

**Authors:** Andressa Wanneska Martins da Silva, Micheline Marie Milward de Azevedo Meiners, Elza Ferreira Noronha, Maria Inês de Toledo

**Affiliations:** ^1^Tropical Medicine, Faculty of Medicine, University of Brasília, Brasília, Brazil; ^2^College of Pharmacy, Faculty of Ceilândia, University of Brasília, Brasília, Brazil

**Keywords:** Chronic Care Model, ACIC, delivery system design, HIV/AIDS, assessment technology, health evaluation

## Abstract

The effectiveness of antiretroviral therapy has rendered HIV infection a manageable chronic condition. Currently, the health systems face the challenge of adopting organizational healthcare models capable of ensuring the delivery of comprehensive care. The Chronic Care Model has been reported for its effectiveness, particularly in terms of delivery system design. In this study, the Assessment of Chronic Illness Care (ACIC) questionnaire, a soft technology widely used for other chronic conditions, was employed on a teaching hospital to evaluate healthcare provided to people living with HIV/AIDS. The ACIC technology is a self-explanatory instrument which diagnoses, among the six components of the Chronic Care Model Framework, areas for quality improvements, indicating at the same time, intervention strategies and achievements. These components are *healthcare network organization, delivery system design, self-management support, decision support, clinical information systems*, and *community.* From May to October 2014, the tool was applied to the multidisciplinary teamwork at the points of care identified, as well as to the hospital management board. Respondents broadly rated care as basic. A pronounced contrast was observed from evaluation by management board and health professional staff in some components like *organization of healthcare* and *clinical information system.* The *self-management support* and *delivery system design* were the components best evaluated by the multidisciplinary team. Combined with the array of services offered, the entry points available at the hospital can ensure healthcare comprehensiveness. However, some gaps were detected, precluding the delivery of an effective care. The ACIC was considered an adequate technology to provide knowledge of the gaps, to promote productive discussions and reflections within teams and to indicate actions to achieve improvements on healthcare for people living with HIV/AIDS.

## Introduction

In 2017, there were an estimated 37 million PLWHA worldwide and 21.7 million people receiving antiretroviral treatment. In Brazil, there were 880 000 registered cases of AIDS, of which more than 100 000 were pregnant women. Preventive measures have been adopted, including post-exposure prophylaxis, testing campaigns, condom distribution to populations at risk, and implementation of national treatment protocols with free provision of drug therapy. At the end of 2017, 87% of PLWHA had been diagnosed and 75% of all diagnosed were already on antiretroviral therapy (Ministério da Saúde [MS], 2017; United Nations Programme on HIV/AIDS [UNAIDS], 2018; World Health Organization [WHO], 2018).

In 1999, Brazil issued the National Policy for Sexually Transmitted Diseases and AIDS (STD/AIDS), containing guidelines and actions for the National Program of STD/AIDS. Objectives, guidelines and priorities were defined from the perspective of the Unified Healthcare System (“*Sistema Único de Saúde*,” SUS) principles – equity, universality, integrality, decentralization and social participation – where the State and society interact in search of health promotion of users. It should be noted that the last three principles sustain SUS, therefore, they must be present in health actions and services (BRASIL, 1999). The National STD/AIDS Program incorporates three coordinated components: (1) Promotion, Protection and Prevention; (2) Diagnosis and Assistance; (3) Institutional Development and Management. Each component is detailed with guidelines, strategies, norms and procedures regarding PLWHA care (BRASIL, 1999).

In 2000, in order to assess the National Policy, the Ministry of Health supported the Qualiaids Research Team to develop and validate a questionnaire, a tool for external assessment based on the Qualiaids Program, as well as its recommendations book, as a monitoring and evaluation mechanism to improve HIV/AIDS, Universidade de São Paulo [USP] (2018). The questionnaire has 84 structure and process indicators and a set of best practice recommendations. The principles and clinical, epidemiological and ethical guidelines of the Program were translated into norms, criteria, indicators and quality standards for the questionnaire elaboration and validation.

Although these efforts have improved prognosis for PLWHA, the challenge to SUS is to adapt the current healthcare model: most PLWHA are retained in the specialized care not being referred to primary care setting. Then, it becomes necessary to change the healthcare model to ensure an effective, comprehensive, multidisciplinary model focused on chronic conditions, aptly integrated with primary healthcare. The traditional model focused in the specialist is unsustainable to the healthcare system (Ministério da Saúde, 2015b).

**FIGURE 1:**
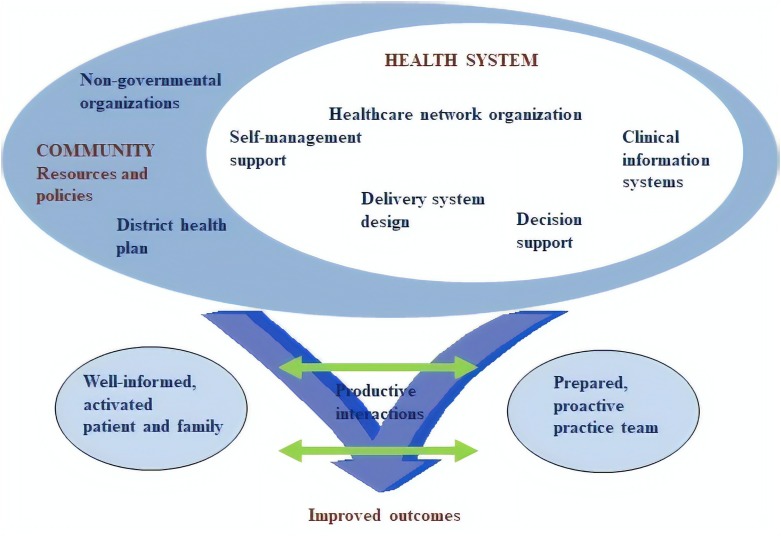
The Chronic Care Model (Wagner, 1998): developed by The MacColl Institute, © ACP-ASIM Journals and Books, reprinted with permission from ACP-ASIM Journals and Books and adapted by authors for SUS.

A global call has been made urging countries to foster research on innovative, optimized management of chronic conditions by healthcare systems, allowing clinical knowledge to be translated to the current healthcare context (World Health Organization [WHO], 2013). The CCM, developed in the United States in the 1990s, identifies six key elements that must function in a coordinated form in order to yield improved healthcare for chronic conditions. These elements are split into two groups: health systems and community (Improving Chronic Illness Care [ICIC], 2018). In Figure 1, we present the CCM model, adapted by us to consider national features from SUS settings, including two additional key elements: *District health plan* and *non-governmental organizations* (Mendes, 2011; Moysés et al., 2012).

In the CCM, changes to the health system should address healthcare network organization, delivery system design, self-management support, decision support, and clinical information systems. The model advocates the establishment of partnerships and the use of resources available in the community to implement the intended changes and align these resources with public policies (Wagner et al., 2005; Mendes, 2011). The CCM implementation can be monitored and evaluated with its own innovative health technology, the ACIC questionnaire (Supplementary Table 1), which diagnoses the situation revealing the nature and degree of the improvements required, indicating intervention strategies and measuring the progress achieved after the interventions (Bonomi et al., 2002; Schwab et al., 2014).

The impact of the CCM on a variety of chronic diseases has been reported, including asthma, diabetes, and depression (Improving Chronic Illness Care [ICIC], 2018). Only seven studies, however, have been retrieved on the application of the CCM to HIV/AIDS (Goetz et al., 2008; Drabo et al., 2010; Tu et al., 2013; Clarke et al., 2015; Mahomed and Asmall, 2015; Massoud et al., 2015; Berenguer et al., 2018). These studies reported improved access and adherence to antiretroviral therapy, implementation of pertinent interventions, and increased involvement of PLWHA with their own care, resulting in clinical, immunological, and virological gains. Of note, a systematic review already published compiled data from 16 papers using CCM Framework for people living with HIV (Pasricha et al., 2013). This systematic review aimed to assess the effectiveness of decision support and clinical information system interventions, examining the outcomes: immunological/virological, medical, psychosocial, economic measures. However, the instruments applied for this assessment were others than ACIC. Therefore, the ACIC remains an innovative approach for HIV/AIDS management. Since our last review in 2018, only one study had, in fact, employed this questionnaire to evaluate HIV care (Drabo et al., 2010).

The purpose of the present investigation was to apply a validated Brazilian Portuguese version of the ACIC questionnaire to diagnose the capacity of care to PLWHA at a Brazilian teaching hospital, bringing the high importance of the CCM Framework as a technology which helps the improvement of the quality of care.

## Materials and Methods

### Study Design, Site and Phases

From May to October 2014, this descriptive study was conducted with the staff of points of care for PLWHA and the management board of a Brazilian teaching hospital – HUB.

The entry points, the points of care, and the delivery system design available for PLWHA were identified by interviewing nutritionists, psychologists, pharmacists, medical interns, and other healthcare professionals. The interviews followed a script comprising three questions concerning PLWHA treated at the HUB: “What are the patient’s entry points?”, “To which points of care is the patient assigned?”, and “To which services is the patient subsequently referred?” In this study, a point of care was defined as any hospital location where PLWHA have their condition directly treated by health professionals (Mendes, 2011).

The term *delivery system design*, defined by Wagner et al. (2005) as *structured reorientation of available healthcare services and interaction between general practitioners and specialists for achieving comprehensive care*, was expressed in our questionnaire as *line of care*, term used in Brazil to represent the reorientation of care flow combined with the relationships that emerge from this flow and are constructed in the light of comprehensiveness (Malta and Merhy, 2010).

In the subsequent phase, the ACIC questionnaire was applied to healthcare professionals at all points of care and to the hospital’s managing board in order to allow comparison of the views held by healthcare professionals and institutional leadership.

### Evaluation Tools and Data Collection

For this investigation was applied an ACIC questionnaire (version 3.5) from MacColl Institute for Health Care Innovation (2010) previously adapted, translated and validated to Brazilian portuguese by Moysés et al. (2012). This validated questionnaire was also adapted by the authors for PLWHA according to the hospital context and terminologies. Since Moysés’ paper applied ACIC to a primary care context and our work take place in the specialized care scenario, necessary adjustments were made. Also, the term “...chronic conditions” was replaced by “... people living with HIV or PLWHA” along the ACIC tool (Moysés et al., 2012). The questionnaire was applied on different dates for 90 min, on average, to a group of at least three healthcare professionals at different points of care. The answers expressed the group consensus. Within the managing board, only the general manager completed the questionnaire. The researchers acted as facilitators and refrained from interfering with discussions or responses.

### How Does the ACIC Questionnaire Work?

The ACIC questionnaire is a self-explanatory instrument which diagnoses, among the six components of CCM Framework, areas for quality improvements, indicating at the same time, intervention strategies and achievements. It covers 35 qualitative indicators divided into seven blocks: one block for each component of the model and an integrative block termed *Integration of CCM components*. These indicators measure processes including technical and interpersonal ones, which can influence the way care is delivered and consequently its success. The interpersonal processes indicators are often ignored because it is not easily available, consisting of limitation in most assessments of quality of care (Donabedian, 1988). In *health system organization*, the questionnaire assesses the management changes that are required to establish a proactive leadership, a healthcare and information flow and incentives to providers and PLWHA. These changes tend to integrate and refine work spaces within the organization (Mendes, 2011; Moysés et al., 2012).

In *delivery system design*, it evaluates how well-defined the tasks are among the health team to ensure a comprehensive and individualized care – adjusted to the social and cultural context of the user. It also measures the system for referral and return-referral which are accountable for linking the points of care (Mendes, 2011).

In *self-management support*, it assesses the knowledge and capability of the user, aiming the patient empowerment – to make decisions, to understand the plan of care and treatment goals; additionally, the service provides emotional support and brings the user to the available community resources, for instance, support groups or peer groups (Epping-Jordan et al., 2004; Wagner et al., 2005; Mendes, 2011; Moysés et al., 2012; Improving Chronic Illness Care [ICIC], 2018).

In *clinical decision support*, the indicators basically focus the use of evidence-based guidelines, training, practical and opportune decisions by the health team, gathering user preferences and health conditions. In addition, the flow of communication between specialists and primary care or interdisciplinary team should improve care (Epping-Jordan et al., 2004; Wagner et al., 2005; Mendes, 2011; Moysés et al., 2012).

In *clinical information system*, it assesses the system of information, including registries, data of individual patients and populations of patients with specific conditions, as well as provides reminders and feedbacks. It should promote, especially, the exchange of information between the various levels of care, leading to a better coordination of information (Epping-Jordan et al., 2004; Wagner et al., 2005; Mendes, 2011; Moysés et al., 2012; Improving Chronic Illness Care [ICIC], 2018).

In *community resources*, it evaluates the implementation of intersectoriality for health, the articulations and partnerships with resources that exist in other sectors of public administration (such as education, sports, social assistance), as well as community organizations (clubs, churches, community centers and support groups such as Alcoholics Anonymous, Narcotics Anonymous, among others) (Wagner et al., 2005). Also, it verifies the District Plan of Health about the resources available to the HIV care (Mendes, 2011; Moysés et al., 2012).

Finally, the ACIC assesses the interrelationship among the six elements of the CCM, linking key elements that contribute to desired clinical and functional outcomes with a positive impact on PLWHA quality of life and health organization effectiveness (Improving Chronic Illness Care [ICIC], 2018).

### Criteria for Analysis

The 35 indicators of ACIC are evaluated individually inside of each block. Each indicator measures, on a scale of 0–11, an institution’s capacity of care provision for chronic conditions. Scores are grouped into four levels: D (limited, 0–2), C (basic, 3–5), B (reasonably good, 6–8), and A (fully developed, 9–11) (Bonomi et al., 2002). ACIC guidelines were followed to analyze the results—i.e., for each completed questionnaire, the mean value of each CCM component was calculated and the average of these means was assigned to the questionnaire. To evaluate PLWHA care, a global score was calculated as the average value of the means obtained at the points of care and management board. Also, a global mean was obtained for each component based solely on the points of care.

The value of each component was analyzed considering the means obtained for each component and applying stratified analysis to identify items exhibiting deficits or limitations.

The Microsoft Office Excel 2013 software was employed for the construction of graphs and analyses.

### Ethics Statement

This study was carried out in accordance with the recommendations of the Resolution 466/12 of the Brazilian Health Council, Research Ethics Committee of the Universidade de Brasília School of Health Sciences with written informed consent from all subjects. The protocol was approved by the Research Ethics Committee of the Universidade de Brasília School of Health Sciences (permit 278.787).

## Results

### Delivery System Design and Points of Care

“What are the entry points of PLWHA?”, “To which points of care are these patients assigned?”, and “To which services are these patients subsequently referred?”

The entry points reported by health professionals were three: the hospital’s emergency service, outpatient pharmacy, and psychosocial support center (termed “*Com-Vivência*”). The identified points of PLWHA care were four: the *Com-Vivência*, the outpatient clinic for IPDs, the outpatient pharmacy, and the inpatient unit for IPDs.

Despite ongoing attempts to certify the HUB as an HIV/AIDS referral center for the Federal District, and particularly for the East Region Healthcare Network, none of the respondents reported return-referrals to other health services. This led us to conclude that patients bear the burden of finding additional healthcare services outside the hospital.

### Assessment of Healthcare to PLWHA at the HUB

**FIGURE 2:**
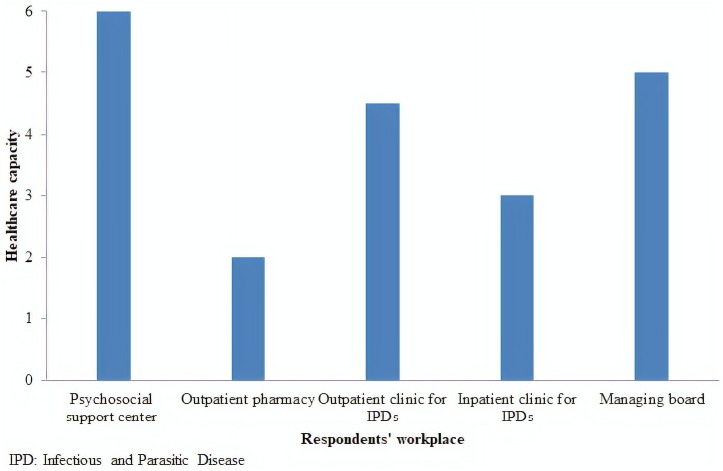
Healthcare capacity as scored with the Assessment of Chronic Illness Care questionnaire by place of work of respondents. Hospital Universitário de Brasília, 2014.

The result of ACIC questionnaire at HUB yielded an overall score of 4 (in the 2–5 range), assigning level C (basic) to the hospital’s capacity of care delivery to PLWHA. The outpatient pharmacy scored lowest (2, level D: limited capacity) (Figure 2).

**FIGURE 3:**
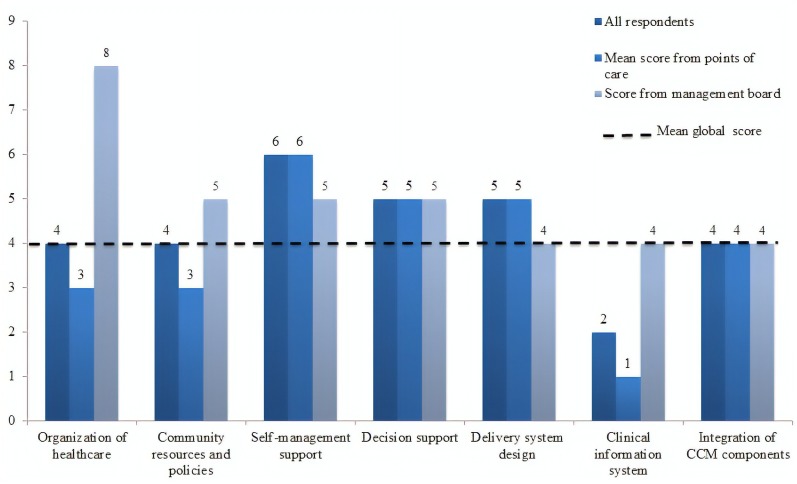
Mean scores for components of the Assessment of Chronic Illness Care questionnaire by type of respondent. Hospital Universitário de Brasília, 2014.

When analyzing the mean scores of ACIC for each component (Figure 3) it was observed that the capacity to employ community resources and policies was rated as basic (mean score 4) by healthcare professionals. The management board acknowledged the importance of the District Healthcare Plan in the care delivery practiced in the HUB. Overall, the coordination between hospital and community resources was regarded as limited. In the view of respondents, the care delivery was not shared between the HUB and community organizations.

A pronounced contrast was observed in the component “Organization of healthcare,” rated as basic (mean, 3) by the point of care staff, but as good (mean, 8) by the managing board (Figure 3). Health professionals acknowledged the role of organizational leadership in effecting changes in PLWHA care. However, they viewed the organizational goals as unclear and the strategies for improvement as restricted to emergency solutions, which are devised and implemented on a case-by-case basis as problems arise. Incentives and regulations for professionals were not employed for PLWHA management purposes.

Next, the components mean scores were analyzed individually to assess how each indicator contributed to the mentioned results. The capacity for self-management support was deemed good (mean score 6) by health professionals. Particularly, they acknowledged the role played by the psychosocial support center in assisting the healthcare team to empower and provide psychosocial support to PLWHA. In general, all the points of care showed engagement with contexts related to treatment adherence, and commitment to seeking suitable solutions for each individual user.

The capacity for clinical decision support was rated as basic (mean score 5) by health professionals who viewed the involvement of other specialists in PLWHA care as limited. Among the healthcare team, continuing education was pursued either by holding weekly meetings to discuss clinical cases and scientific papers or at the personal initiative of staff members, often without acknowledgment from the managing board. The Ministry of Health rarely provided updates or refresher courses on national clinical guidelines. PLWHA had access to information on clinical guidelines (verbally or in the form of educational materials) only upon request. The Com-Vivência center continually added this information to the strategies for self-management support.

Delivery system design was rated as basic (mean score 5) by health professionals. The *Com-Vivência* and emergency service were described as the principal entry points. Although PLWHA can use the entire range of services available from the hospital, their delivery system design takes place primarily at the points of care identified, which, however, are not coordinated for multiprofessional teamwork. There is a chief of staff who heads each of these services, but leadership was not clearly perceived by respondents. A medical appointment management system is currently in operation, and periodic appointments with a single specialist are given priority. PLWHA monitoring complies with clinical guidelines or is tailored to the patient’s needs, being mostly performed by the outpatient clinic for IPDs and the *Com-Vivência* center. Programmed care was only available for complications or when requested by users. Because neither referral nor return-referral system is in operation, it has been dealt with in a non-standardized, case-by-case manner. Communication between points of care was poor.

Health professionals assigned the lowest score to the clinical information system, evaluated as limited (mean score 1), since the HUB has no electronic outpatient registry or outpatient medical record system. In fact, each point of care has its own record system—paper-based, except at the inpatient unit for IPDs. The outpatient pharmacy employs an electronic system for drug dispensing control, managed by the Ministry of Health, but does not keep clinical records. Pharmacy staff has access only to data retrievable from medical records or directly informed by users. The healthcare team has standardized a care delivery plan for PLWHA.

**FIGURE 4:**
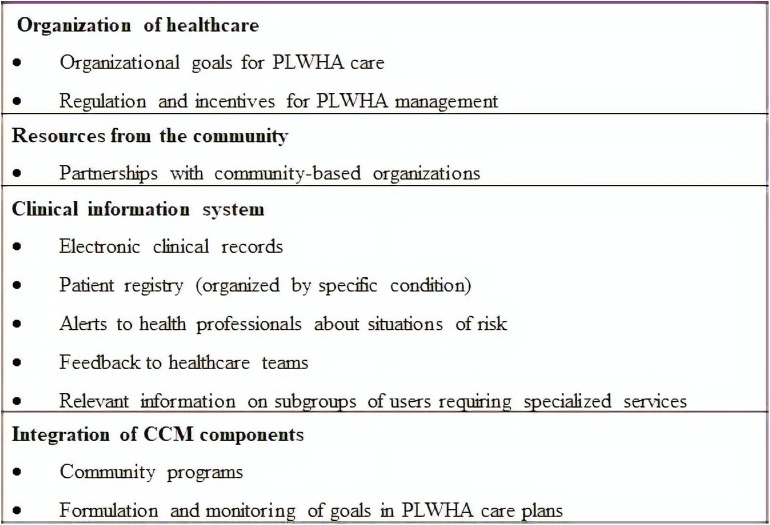
Assessment of Chronic Illness Care indicators rated as having limited healthcare capacity. Hospital Universitário de Brasília, 2014.

The indicators rated as limited (ACIC mean score ≤ 2) are shown in Figure 4, based on a stratified analysis of all 35 ACIC indicators.

## Discussion

The ACIC is a comprehensive tool designed to represent poor to optimal healthcare management and support, assessing technical and interpersonal processes which may influence the quality of care (Donabedian, 1988). It may be applied to all chronic conditions or constellations of conditions (Bonomi et al., 2002; Hibbard and Greene, 2013). The proposal of ACIC being applied for HIV/AIDS care in Brazil is innovative and it fulfills an important gap in the assessment of quality of care. Despite of being an external tool for assessment of quality of care, Qualiaids is not able to measure the nuances of interpersonal processes. For this, both questionnaires, ACIC and Qualiaids, could support the improvement of quality of care for PLWHA, as the ACIC tool complements the Qualiaids as a self-assessment tool in the perspective of improving results.

Moreover, the application of ACIC is fast and each indicator facilitates discussion, converging to a consensus. The highest score describes the optimal practice, situating the best position that an organization could reach during the diagnoses, the intervention or the assessment (Bonomi et al., 2002; Moysés et al., 2012). Therefore, this questionnaire quickly highlights which areas of the healthcare need to be improved, delivers guidance along this process and monitors progress over time in order to promote a comprehensive care (Bonomi et al., 2002; Drabo et al., 2010; Schwab et al., 2014).

The ACIC proved useful as a soft technology for the situational diagnosis of healthcare delivered to PLWHA at the teaching hospital in the Brazilian setting. The questionnaire fostered discussions within the healthcare team, encouraging its members to actively seek approaches for improvement.

The self-assessment of the hospital’s capacity of care delivery to PLWHA, rated as basic (mean score 4), was the main finding emerging from the ACIC instrument, revealing that several aspects need to be improved for a proper management addressing chronic conditions. A literature survey retrieved a single study that applied the complete ACIC to HIV/AIDS care. The study, by Drabo et al. (2010), comprised three hospitals and eight healthcare centers randomly selected from three districts in Burkina Faso and yielded a mean score of 4, assigning basic capacity to PLWHA care (Drabo et al., 2010). In both studies, healthcare system organization was rated as basic. In the present study, the organizational goals, strategies to improve healthcare delivery, and regulation and incentives to professionals, all of which were rated lowest, were the indicators that most influenced the global result.

The differences observed in the perceptions held by health professionals and managing board indicate the need for greater transparency in leadership responsibilities and organizational strategies for PLWHA care. Institutional goals and plans were viewed as poorly defined, a feature that can undermine the motivation of health professionals (Wagner et al., 2001). Proactive leaderships, capable of establishing rapport with team members, are associated with a more positive stance in the workplace and greater commitment of staff, translating to consistent, effective changes in care delivery (Wagner et al., 2001; Benzer et al., 2011). Lack of interest and poor commitment of team leaders, absence of committed professionals, and the unavailability of updated information technologies seem to negatively impact CCM implementation. Having contradictory results is intrinsic of ACIC questionnaire, that is why it should be applied periodically (at least once every 6 months), from the perspective that with each new assessment the results will come closer to reality in order to improve quality of care (Improving Chronic Illness Care [ICIC], 2018). Further studies to clarify conditions predictive of CCM success are warranted (Coleman et al., 2009; Davy et al., 2015).

Both studies highlighted low or ineffective use of community resources. In our study an interaction of the health service with the community resources and non-governmental organizations were highly limited. However, Drabo et al. (2010) reported that, despite the absence of formal partnership arrangements, the institutions investigated worked with community-based organizations, promoting joint efforts, with gains for PLWHA.

Using community resources minimizes duplication of effort, reduces healthcare system costs, and raises the quality of care delivered (Wagner et al., 2005; Mendes, 2011).

On the other hand, in both studies the best rating was assigned to self-management support, for its emphasis on providing advice at treatment outset, combined with individual appointments and peer group support. Studies have shown promising results of interventions designed to promote self-management and user empowerment, even when support is provided via telephone calls (Wagner et al., 2001; Damush et al., 2010). Interventions should be user-centered, provide support and health education to enhance the user’s ability in the management of their condition. Additionally, psychosocial support to users and their families should be offered (Damush et al., 2010; Malta and Merhy, 2010; Tu et al., 2013).

Both clinical decision support and delivery system design were rated as basic by Drabo et al. (2010) and likewise in the present study. Poor communication between specialists and other professionals, including primary care physicians, characteristic of the current referral and return-referral system, was perceived as a hurdle to be overcome. A comprehensive care requires smoother communication among all providers to accomplish an individualized therapeutic plan (Barceló et al., 2010; Mendes, 2011).

At the teaching hospital, clinical decision support to PLWHA followed the *Clinical protocol and therapeutic guidelines for management of HIV infection*, in compliance with recommendations of the Ministry of Health (Ministério da Saúde, 2015a). In contrast with the obstacles to the provision of antiretroviral therapy reported for Burkina Faso (Drabo et al., 2010), Brazil ensures free access to antiretroviral therapy to all PLWHA, managed through the National Medication Logistics Control System. This contrast reveals a weakness of the ACIC instrument in the evaluation of a crucial measure, the access of PLWHA to drug therapy, since the questionnaire assigned the same score to the two very different policies adopted by each respective country.

While Drabo et al. (2010) reported a lack of health professionals, the present study revealed obstacles in multidisciplinary training and in communication across points of care. The aggregation of pharmacists, nurses, and social workers to the service network and the promotion of communication across points of care as routine are expected to decrease teamwork fragmentation, ultimately allowing the monitoring of users, to meet the needs of this population. Multiprofessional teamwork has been associated with positive functional outcomes in users with chronic conditions (Bodenheimer et al., 2009; Carter et al., 2009).

The clinical information system was critical in both studies. Incomplete and paper-based records not only have a detrimental effect on the management of interventions, but also preclude reliable evaluation of the quality of care delivered, increasing the likelihood of medical error (Hillestad et al., 2005; Chaudhry et al., 2006; Kalogriopoulos et al., 2009). Safety can be increased with the use of electronic clinical records, as well as by employing more low-tech resources such as reminders, alerts, brochures, and letters tailored for users. Electronic prescriptions, combined with ready access to clinical information from different services and points of care, contribute toward comprehensiveness in care delivery. The benefits of clinical information systems have been observed in health promotion efforts and in the prevention of complications and risk factors (Hillestad et al., 2005; Chaudhry et al., 2006; Carter et al., 2009; Kalogriopoulos et al., 2009). However, these systems typically require large investments in material and human resources to become efficient and can often be met with resistance by health professionals. Nonetheless, clinical information systems can be convenient facilitating tools, although insufficient to transform the healthcare system by themselves (Tomasi et al., 2004; Hillestad et al., 2005; Kalogriopoulos et al., 2009).

Some of observed limitations about the ACIC questionnaire should be considered. It is a technology that qualifies but does not describe all the pieces of evidence related to care – as structure, process and outcomes at Donabedian evaluation; in this sense, it must be analyzed according to the local context and other supportive data. Importantly, this tool is not a “step-by-step,” detailed evaluation about the care process, but it provides the pillars to reach high quality of care (Bonomi et al., 2002; Drabo et al., 2010). The consensus method is important to gather every opinion and summarize them in only one. However, it could conceal biases because of the opinion coming from a person in leadership role during the process. For this, we stratified datareal to board manager and health professionals. Besides, in the first assessment, we could observe that the teams frequently over- or underestimate the quality of care as a result from the misperception of the care they are providing. However, during the CCM Framework implementation process the teams notice what effective care is and their scores could decrease or increase depending on their recently acquired knowledge. When the capability of comprehensive care increases and teams continue implementing effective changes, these scores tend to be improved. Of note, most studies that applied CCM Framework and ACIC addressed a variety of chronic conditions other than HIV/AIDS (Mendes, 2011; Schwab et al., 2014). Therefore, there is a scarce evidence for HIV assessment with this technology, allowing mild consistency about the strengths and limitations in the tool application.

## Conclusion

Despite the limitations, we considered that the ACIC succeeds to evaluate the key components for a comprehensive healthcare, encourages reflection from the healthcare team at the HUB, generates helpful discussions, raises awareness among the professionals overwhelmed with service routines, and indicates goals to be pursued to improve the quality of healthcare for PLWHA.

In summary, the ACIC technology proved useful for the situational diagnosis of healthcare delivery to PLWHA at a teaching hospital in Brazil. ACIC concomitant application with Qualiaids provides interpersonal processes indicators, often disregarded in most assessments, which would improve the PLWHA quality of care. Additional aspects to be explored include the ACIC use in other settings, interventions evaluations and monitoring and the CCM implementation at institutions that provide healthcare improvement to PLWHA.

## Author Contributions

AS and MM designed this work, drafted, and reviewed the manuscript. MT and EN reviewed the draft. All authors approved the manuscript for publication and agreed to be accountable for all aspects of this work.

## Conflict of Interest Statement

The authors declare that the research was conducted in the absence of any commercial or financial relationships that could be construed as a potential conflict of interest.

## Abbreviations

ACIC: Assessment of Chronic Illness Care
CCM: Chronic Care Model
HUB: Hospital Universitário de Brasília
IPD: infectious and parasitic disease
PLWHA: people living with HIV/AIDS
SUS: Unified Health System
