# Supplementary material for: The Use of Assessment of Chronic Illness Care Technology to Evaluate the Institutional Capacity for HIV/AIDS Management
Source: Front Pharmacol. 2019 Feb 27;10:165. doi: 10.3389/fphar.2019.00165 (PMC6400989; doi:10.3389/fphar.2019.00165)
Supplement: Supplementary file 1 [file Table_1.DOCX]

##### Avaliação da Capacidade Institucional para a Atenção às Condições Crônicas – PVHA (ACIC – versão 3.5)

*“As pessoas necessitam de um cuidado que torne-as*

*capacitadas para manejar seus problemas de saúde crônicos.*

*Elas precisam de apoio para fazer coisas que gostam, para seguir seu*

*tratamento e manter-se fisicamente ativas. Estamos focados em criar*

*no sistema de saúde a habilidade para ajudar os pacientes nestes objetivos”*

Michael Von Korff, ScD, pesquisador senior no CHS

##### **Introdução**

Os sistemas de saúde (instituições ou unidades de saúde) requerem ferramentas práticas de avaliação para guiar os esforços de melhoria da qualidade e para avaliar mudanças feitas na atenção às condições crônicas. Na resposta a esta necessidade, a equipe de funcionários ICIC desenvolveu o questionário de Avaliação da Capacidade Institucional para a Atenção às Condições Crônicas (Assessment of Chronic Illness Care - ACIC) (Bonomi e outros., 2002). Elaborado a partir de uma ferramenta desenvolvida pelo Serviço de Saúde Indiano para a avaliação da atenção ao diabetes (Acton e outros., 1993, 1995), o ACIC é proposto para ser utilizado por equipes de saúde para: (1) identificar áreas para a melhoria da atenção em condições crônicas antes da implementação de ações/ projetos de melhoria de qualidade, e (2) avaliar o nível e a natureza das melhorias feitas em resposta às intervenções adotadas.

O questionário ACIC derivou de intervenções específicas, baseadas em evidência, para os seis componentes do modelo de atenção para condições crônicas, conforme figura abaixo (recursos da comunidade, organização do sistema de saúde, apoio para o autocuidado, desenho da linha de cuidado, suporte para decisões clínicas e sistema de informações clínicas) (Wagner, 1998). Como o modelo, o ACIC aborda os elementos básicos para melhorar o cuidado a estas condições na comunidade, no sistema de saúde (instituições e unidades), na prática clínica e no nível do usuário.

**Instruções para o preenchimento**

Este instrumento foi desenhado para o monitoramento da capacidade institucional de uma rede de atenção à saúde ou de algum ponto de atenção, por exemplo, um Hospital ou uma Unidade de Atenção Primária à Saúde, para desenvolver o Modelo de Atenção a Condições Crônicas. O resultado pode apoiar gestores e equipes de saúde a melhorar a atenção às condições crônicas.

Após uma leitura do questionário e uma breve discussão sobre seu conteúdo, ele deve ser preenchido por um profissional de saúde ou, preferencialmente, em conjunto por uma equipe de saúde de um mesmo local.

1. **Responda cada questão** na perspectiva de um local (ex. UBS, hospital, clinica especializada) que atua na atenção às condições crônicas.

Indique o nome e tipo de local em análise:_______________________________

1. **Responda cada questão** explicitando como sua organização está atuando frente a uma doença ou condição.

Especifique a doença ou condição____________________________________

1. Descreva brevemente o processo utilizado para preencher o questionário (p. ex., preenchido por consenso em reunião presencial; preenchido por um líder em consulta com outros membros da equipe; preenchido por cada membro da equipe em separado e feito uma média das respostas).

Descrição:_______________________________________________________________________________________________________________________________________________________________________________________

1. Cada linha desse questionário apresenta aspectos fundamentais da atenção às condições crônicas. Cada aspecto é dividido em níveis e em valores que demonstram os vários estágios na melhoria da atenção às condições crônicas. Os estágios são representados pelos níveis D, C, B ou A e os valores de 0 a 11. Os valores mais baixos expressam capacidades institucionais menores e os mais altos capacidades institucionais maiores ou totalmente desenvolvidas para a atenção às condições crônicas. Para cada linha, **identifique o nível e então, dentro desse nível, circule o valor** que melhor descreve o nível de atenção praticado na instituição em análise em relação às condições crônicas consideradas. Caso exista divergência no grupo, discutam até chegar a um consenso. Apenas um valor pode ser preenchido por linha.
2. **Some o total dos valores de cada seção** e calcule a pontuação média correspondente. Preencha os campos no final de cada seção com os valores obtidos. Então, na última folha, preencha os campos com os valores obtidos de cada seção. Some todas as pontuações e complete a pontuação média para o programa como um todo.

**Avaliação da Capacidade Institucional para a Atenção às Condições Crônicas – PVHA (ACIC)**

**Parte 1: Organização da Atenção à Saúde: A estruturação da gestão de programas (ou políticas institucionais) para** condições crônicas pode ser mais efetiva se todo o sistema (organização, instituição ou unidade) no qual a atenção é prestada esteja orientada e permita maior ênfase no cuidado às condições crônicas.

| **Componentes** | **Nível D** | **Nível C** | **Nível B** | **Nível A** |
| --- | --- | --- | --- | --- |
| **A liderança organizacional (direção do HUB) em relação à mudanças na atenção às PVHA...** | …não existe ou há pouco interesse. | …está refletido na visão, na missão e no plano estratégico, mas não há recursos comprometidos para a execução do trabalho. | …está incorporado na organização e na sua liderança superior, com recursos específicos comprometidos (financeiros e humanos). | …é parte de planos estratégicos de longo prazo, há recursos humanos e financeiros comprometidos e possui equipe técnica designada, que é responsável pelas ações. |
| **Pontuação** | 0 1 2 | 3 4 5 | 6 7 8 | 9 10 11 |
| **Metas**  **organizacionais para**  **a atenção às PVHA...** | …não existem ou são muito limitadas | …existem, estão documentadas, mas não são revisadas regularmente. | … existem, estão documentadas, são monitoradas e revistas. | … existem, estão documentadas, são monitoradas e revistas periodicamente e estão incorporadas nos planos de melhoria de qualidade institucional. |
| **Pontuação** | 0 1 2 | 3 4 5 | 6 7 8 | 9 10 11 |
| **Estratégias para a**  **melhoria da atenção**  **às PVHA...** | …são informais e não são organizadas ou apoiadas de forma consistente. | …utilizam soluções emergenciais para resolver pontualmente os problemas que se apresentam. | …utilizam estratégias efetivas quando surgem problemas. | …utilizam estratégias efetivas, usadas pró-ativamente para garantir que se alcancem as metas organizacionais. |
| **Pontuação** | 0 1 2 | 3 4 5 | 6 7 8 | 9 10 11 |
| **Regulação e incentivos para a atenção às PVHA...** | …não são utilizados para definir ou pactuar as metas de desempenho clínico. | …são usados para controlar a  utilização de serviços e custos na atenção às PVHA. | …são usados como estratégia para apoiar nas metas de atenção aos usuárias. | …são usados para motivar e empoderar os profissionais da saúde e apoiar no alcance das metas de desempenho clínico. |
| **Pontuação** | 0 1 2 | 3 4 5 | 6 7 8 | 9 10 11 |
| **Lideranças institucional...** | …não estimulam a atenção às PVHA. | …não dão prioridade à melhoria da atenção às PVHA. | …encorajam esforços para a  melhoria da atenção às PVHA. | …priorizam as ações e participam dos esforços para a melhoria da atenção às PVHA. |
| **Pontuação** | 0 1 2 | 3 4 5 | 6 7 8 | 9 10 11 |
| **Benefícios e incentivos ao usuário (econômicos e morais)...** | …desencorajam o autocuidado ou as mudanças do sistema de assistência ao usuário. | …nem encorajam nem desencorajam o autocuidado e as mudanças do sistema de assistência ao usuário. | …encorajam o autocuidado ou as mudanças do sistema dpara a assistência ao usuário. | …são especificamente desenhados para promover a integralidade e melhor atenção às PVHA. |
| **Pontuação** | 0 1 2 | 3 4 5 | 6 7 8 | 9 10 11 |

Pontuação Total para Organização da Atenção à Saúde ________ Pontuação Média (Pontuação Total para Organização da Atenção à Saúde / 6)_________

**Parte 2: Articulação com a comunidade:** Articulação entre o sistema de saúde (instituições ou unidades de saúde) e os recursos comunitários tem um importante papel na gestão/ manejo de condições crônicas.

| **Componentes** | **Nível D** | **Nível C** | **Nível B** | **Nível A** |
| --- | --- | --- | --- | --- |
| **Articulação das instituições de saúde com os recursos comunitários, para participação dos usuários...** | …não é feita sistematicamente. | …é limitada a uma lista de recursos comunitários identificados em um formato acessível. | … é realizada por meio de pessoal designado para assegurar que os usuários e os profissionais da saúde utilizem ao máximo os  recursos comunitários. | …é realizada por meio de ativa coordenação entre a instituição de saúde, as organizações comunitárias e os usuários. |
| **Pontuação** | 0 1 2 | 3 4 5 | 6 7 8 | 9 10 11 |
| **Parcerias com organizações comunitárias...** | …não existem. | …estão sendo consideradas, mas não foram implementadas. | …estão estruturadas para apoiar programas de atenção às condições crônicas na instituição. | …são ativas e formalmente estabelecidas para dar suporte aos programas de atenção às condições crônicas da instituição e da comunidade. |
| **Pontuação** | 0 1 2 | 3 4 5 | 6 7 8 | 9 10 11 |
| **Plano Distrital de Saúde (ou outras resoluções do Conselho Distrital de Saúde ou de instância de representação da comunidade)...** | …não aborda em seu contexto recursos, medidas ou direitos dos usuários referentes à atenção às PVHA. | …aborda orientações sobre recursos, medidas ou direitos das PVHA quanto a atenção à saúde, mas não foram implantadas, pois tem função apenas cartorial. | …aborda orientações sobre recursos, medidas ou direitos das PVHA quanto a atenção à saúde; acompanha a programação da instituição de saúde relativa às PVHA. | …aborda orientações sobre recursos, medidas ou direitos das PVHA quanto a atenção à saúde; acompanha pró-ativamente a programação da instituição de saúde relativa às condições crônicas, controlando os incentivos de desempenho das equipes. |
| **Pontuação** | 0 1 2 | 3 4 5 | 6 7 8 | 9 10 11 |

Pontuação Total para Articulação com a Comunidade ______ Pontuação Média (Pontuação total para Articulação com a Comunidade / 3) _________

**Nível Prático da Atenção.** Muitos componentes da atenção às condições crônicas que acontecem no nível das Unidades/ Serviços de Saúde (consultórios, clínicas, hospitais, etc.) têm demonstrado potencial para melhorar a qualidade da atenção prestada. Estes componentes englobam áreas como o apoio para o autocuidado, desenho da linha de cuidado (a partir da rede de atenção à saude e integração dos pontos de atenção), suporte às decisões clínicas e os sistemas de informação clínica.

------------------------------------------------------------------------------------------------------------------------------------------------------------

**Parte 3: Autocuidado apoiado.** O autocuidado apoiado efetivo pode ajudar as pessoas com condições crônicas e suas famílias a lidar com os desafios de conviver e tratar a condição crônica, além de reduzir as complicações e sintomas da doença.

| **Componentes** | **Nível D** | **Nível C** | **Nível B** | **Nível A** |
| --- | --- | --- | --- | --- |
| **Registro e Avaliação das atividades e necessidades de autocuidado apoiado...** | …não são realizadas. | …estão sendo planejadas. | …são realizadas de maneira padronizada. | …são realizadas de maneira padronizada e articuladas com o plano de cuidado de cada usuário (p.ex. plano terapêutico, prontuário). |
| **Pontuação** | 0 1 2 | 3 4 5 | 6 7 8 | 9 10 11 |
| **Suporte para o autocuidado apoiado ...** | …é limitado a distribuição de  informação (panfletos, folders e outras informações escritas). | …é disponibilizado por meio de atividades educacionais em grupo ou individual sobre autocuidado apoiado. | …é oferecido por educadores em saúde capacitados, que são designados para orientar o autocuidado apoiado, que fazem parte de equipe multiprofissional de saúde, mas envolve apenas as PVHA referidas. | …é oferecido por educadores em saúde especialmente capacitados em metodologias de empoderamento e de resolução de problemas, envolvendo a maioria das PVHA |
| **Pontuação** | 0 1 2 | 3 4 5 | 6 7 8 | 9 10 11 |
| **Suporte psicossocial às PVHA e seus familiares...** | ...não é realizado de forma consistente. | …é realizado para pessoas usuárias ou famílias específicas por meio de referência. | … é estimulado e disponibilizado por meio de grupos de pares e atenção em grupos específicos. | …é parte integrante da atenção às PVHA e inclui avaliação sistemática, com envolvimento contínuo em grupos de pares e atenção em grupo. |
| **Pontuação** | 0 1 2 | 3 4 5 | 6 7 8 | 9 10 11 |
| **Intervenções efetivas**  **de mudança de**  **comportamento de pessoas usuárias e de suporte de pares ou grupos de apoio...** | …não estão disponíveis. | …limitam-se a distribuição de informação (panfletos, folders e outras informações escritas). | ...estão disponíveis somente por meio de referência a centros especializados com pessoal capacitado. | …estão disponíveis prontamente e fazem parte integral da rotina da atenção às PVHA e utilizam rotineiramente tecnologias de mudança de comportamento (modelo transteórico de mudanças, entrevista motivacional, grupo operativo, processo de solução de problemas). |
| **Pontuação** | 0 1 2 | 3 4 5 | 6 7 8 | 9 10 11 |

Pontuação Total para Apoio ao Autocuidado _______ Pontuação Média (Pontuação Total para Apoio ao Autocuidado / 4) _______

**Parte 4: Suporte à Decisão Clínica.** O manejo efetivo de condições crônicas assegura que os profissionais de saúde tenham acesso a informações baseadas em evidência para apoiar as decisões (diagnóstico, prognóstico, tratamento) na atenção às pessoas usuárias. Isto inclui diretrizes e protocolos baseados em evidência, adaptado ao serviço de saúde, que capacitem aos profissionais e garantam o envolvimento dos usuários, de forma a tornar as equipes multiprofissionais de saúde capazes de identificar estratégias efetivas de cuidado.

| **Componentes** | **Nível D** | **Nível C** | **Nível B** | **Nível A** |
| --- | --- | --- | --- | --- |
| **Diretrizes clínicas baseadas em evidência...** | …não estão disponíveis. | …estão disponíveis, mas não são integradas à atenção às PVHA | … estão disponíveis e integradas às atividades de educação permanente dos profissionais de saúde. | …estão disponíveis, implantadas por educação permanente dos profissionais e integradas à atenção às PVHA por meio de recordatórios, feedbacks e incentivos para sua adoção pelos profissionais de saúde. |
| **Pontuação** | 0 1 2 | 3 4 5 | 6 7 8 | 9 10 11 |
| **Envolvimento de**  **especialistas no apoio à atenção primária à saúde...** | …é feito por meio de referenciamento tradicional. | …é alcançado por meio da participação de especialistas, para aumentar a capacidade do sistema de atenção à saúde, implementando rotineiramente as diretrizes clínicas. | …inclui a participação de liderança de especialistas no processo de educação permanente das equipes de atenção primária em saúde (treinamento em serviço). | …inclui a participação de especialistas que participam do processo de educação permanente dos profissionais da atenção primária à saúde, de sessões de discussão de casos clínicos e trabalho clínico conjunto. |
| **Pontuação** | 0 1 2 | 3 4 5 | 6 7 8 | 9 10 11 |
| **Educação permanente dos profissionais de saúde para a atenção às PVHA...** | …é feita esporadicamente. | …é feita sistematicamente por meio de métodos tradicionais de educação. | …é feita sistematicamente utilizando métodos educacionais adequados para a mudança de comportamento de adultos para uma parte dos profissionais de saúde, com base nas diretrizes clínicas. | …é feita sistematicamente e inclui toda a equipe envolvida na atenção às PVHA, utilizando métodos educacionais adequados para a mudança de comportamento de adultos, com base nas diretrizes clínicas, e envolve métodos como comunicação interpessoal e apoio ao autocuidado. |
| **Pontuação** | 0 1 2 | 3 4 5 | 6 7 8 | 9 10 11 |
| **Informação às PVHA sobre as diretrizes clínicas...** | …não é realizada. | …é realizada quando a pessoa usuária solicita ou por meio de publicações. | …é realizada para pessoas usuárias específicas, por meio de material educativo para cada diretriz clínica. | …é realizada para a maioria das PVHA, por meio de métodos adequados de educação em saúde, específicos para cada diretriz clínica e inclui a descrição do papel do usuário para obter sua adesão à diretriz clínica |
| **Potuação** | 0 1 2 | 3 4 5 | 6 7 8 | 9 10 11 |

Pontuação Total para Suporte às Decisões ____ Pontuação Média (Pontuação Total para Suporte às Decisões /4) _____

**Parte 5: Desenho da Linha de Cuidado.** A evidência sugere que a gestão efetiva da atenção às condições crônicas envolve mais que a simples adição de intervenções a um sistema focado no cuidado de condições agudas ou de urgência. São necessárias mudanças na organização do sistema, realinhando a oferta do cuidado às necessidades do usuário.

| **Componentes** | **Nível D** | **Nível C** | **Nível B** | **Nível A** |  |
| --- | --- | --- | --- | --- | --- |
| **Trabalho em equipe...** | …não existe. | …é realizado por meio da disponibilização de profissionais com treinamento apropriado em cada um dos pontos de atenção às PVHA. | …é assegurado por meio de  reuniões regulares das equipes,  com enfoque nas diretrizes clínicas, nos papéis e responsabilidades de cada membro da equipe e nos problemas da atenção às PVHA. | …é assegurado por meio de equipes que se reúnem regularmente, têm atribuições bem definidas, o que inclui educação para o autocuidado apoiado, o monitoramento pró-ativo dos usuários e recursos de coordenação da atenção às PVHA. | |
| **Pontuação** | 0 1 2 | 3 4 5 | 6 7 8 | 9 10 11 | |
| **Liderança das**  **Equipes de Saúde…** | …não é reconhecida localmente nem pela instituição de saúde. | …é assumida pela instituição e possui no orgranograma papéis organizacionais específicos. | …é assumida pela instituição por meio da designação de um líder, mas seu papel na atenção às PVHA não está definido. | …está garantida pela instituição por designação de um líder que garante a definição clara dos papéis e responsabilidades de cada membro da equipe na atenção às PVHA. | |
| **Pontuação** | 0 1 2 | 3 4 5 | 6 7 8 | 9 10 11 | |
| **Sistema de**  **agendamento...** | … não está organizado. | …está organizado só para o atendimento de urgência (agudas) e algumas situações individuais programadas das PVHA. | …está organizado e inclui agendamentos para consultas individuais periódicas e atenção em grupo das PVHA. | …está organizado e inclui agendamentos para consultas individuais e atenção em grupo, facilitando o contato com diferentes profissionais em uma única visita. | |
| **Pontuação** | 0 1 2 | 3 4 5 | 6 7 8 | 9 10 11 | |
| **Monitoramento das PVHA…** | …não está organizado. | …é programado de acordo com as diretrizes clínicas. | …é assegurado pelos profissionais, de acordo com a necessidade do usuário. | …é organizado pela equipe de saúde e está adaptado às necessidades dos usuários, variando em intensidade e metodologia (telefone, contato pessoal, em grupo, e-mail), com base nas diretrizes clínicas. | |
| **Pontuação** | 0 1 2 | 3 4 5 | 6 7 8 | 9 10 11 | |
| **Atenção programada para as PVHA...** | …não está disponível. | …é utilizada ocasionalmente para usuários com complicações. | …é uma opção para usuários que se interessam por este tipo de atenção. | …é utilizada para a maioria dos usuários, incluindo monitoramento regular, intervenções preventivas e autocuidado apoiado, podendo ser consulta individual ou em grupo. | |
| **Pontuação** | 0 1 2 | 3 4 5 | 6 7 8 | 9 10 11 | |
| **Referência e contrarreferência ou acesso a serviços de apoio diagnóstico às PVHA...** | …não é uma prioridade. | …depende de comunicação  escrita entre profissionais da atenção primária e especialistas ou outros serviços. | …é uma prioridade, mas não está implementada sistematicamente. | …é uma alta prioridade e as intervenções incluem uma coordenação ativa entre a atenção primária, os especialistas ou outros serviços ou pontos de atenção. | |
| **Pontuação** | 0 1 2 | 3 4 5 | 6 7 8 | 9 10 11 | |

Pontuação Total para Desenho da Linha de Cuidado ____Pontuação Média (Pontuação Total para Desenho da Linha de Cuidado/ 6) _______

**Parte 6: Sistema de Informação Clínica.** Informação útil e oportuna, individualizada por usuário e por subpopulações de usuários com condições específicas, é um aspecto crítico de modelos de atenção efetivos, especialmente aqueles que empregam abordagens populacionais.

| **Componentes** | **Nível D** | **Nível C** | **Nível B** | **Nível A** |
| --- | --- | --- | --- | --- |
| **Prontuário clínico eletrônico...** | …não está disponível. | …está disponível, mas é construído individualmente. | …está disponível e é construído com base familiar. | …está disponível, é construído com base familiar e coerente com as diretrizes clínicas e com o modelo de atenção às PVHA |
| **Pontuação** | 0 1 2 | 3 4 5 | 6 7 8 | 9 10 11 |
| **Registro das pessoas usuárias (lista de PVHA por estrato de risco)...** | …não está disponível. | …está disponível, inclui nome, diagnóstico, informação de contato e data da última consulta, em papel ou banco de dados informatizado. | …está disponível, permite identificar subpopulações por estratificação de risco, segundo as diretrizes clínicas. | … está disponível, permite identificar subpopulações por estratificação de risco segundo as diretrizes clínicas e permite emitir alertas e lembretes sobre atendimentos necessários ou pendências. |
| **Pontuação** | 0 1 2 | 3 4 5 | 6 7 8 | 9 10 11 |
| **Alertas para os profissionais...** | …não estão disponíveis. | …estão disponíveis, incluem a notificação geral da existência de condições crônicas, mas não definem os tipos de intervenções necessárias no momento do atendimento. | …estão disponíveis, incluem indicação de intervenções necessárias por subpopulações em relatórios periódicos e definem intervenções necessárias no momento do atendimento. | … estão disponíveis, incluem indicação de intervenções necessárias por subpopulações em relatórios periódicos e dão informações específicas para as equipes estimular sua adesão às diretrizes clínicas no momento do atendimento. |
| **Pontuação** | 0 1 2 | 3 4 5 | 6 7 8 | 9 10 11 |
| **Feedbacks para a equipe de saúde...** | …não estão disponíveis. | …são fornecidos em intervalos irregulares e de forma impessoal. | …ocorrem em intervalos suficientes para monitorar o desempenho e são específicos para cada equipe. | …são fornecidos oportunamente, específicos para uma determinada equipe, de forma pessoal e rotineira por um líder para melhorar o desempenho da equipe. |
| **Pontuação** | 0 1 2 | 3 4 5 | 6 7 8 | 9 10 11 |
| **Informação sobre subgrupos relevantes de pessoas usuárias...** | …não está disponível. | …está disponível, mas somente pode ser obtida com esforços especiais ou programação adicional. | …está disponível eventualmente, mas só pode ser obtida quando solicitada. | …está disponível rotineiramente para os profissionais de saúde para ajudá-los no planejamento do cuidado. |
| **Pontuação** | 0 1 2 | 3 4 5 | 6 7 8 | 9 10 11 |
| **Plano de cuidado das pessoas usuárias...** | …não é elaborado. | …é elaborado em formato padrão pela equipe de saúde. | …é feito em conjunto pela equipe de saúde e pelo usuário e inclui o autocuidado e metas terapêuticas. | …é feito em conjunto pela equipe de saúde e pelo usuário, inclui metas terapêuticas e ações de autocuidado, sendo monitorado regularmente. |
| **Pontuação** | 0 1 2 | 3 4 5 | 6 7 8 | 9 10 11 |

Pontuação Total para Sistema de Informação Clínica ____Pontuação Média (Pontuação Total para Sistema de Informação Clínica / 6) ________

**Parte 7. Integração dos Componentes do Modelo de Atenção às Condições Crônicas.** Sistemas de saúde efetivos integram e combinam todos os elementos do modelo, por exemplo, associando as metas de autocuidado com os registros nos sistemas de informação, ou associando políticas locais com atividades dos planos de cuidado dos usuários (locais para desenvolvimento de atividades físicas, estruturação de hortas comunitárias, etc.).

| **Componentes** | **Nível D** | **Nível C** | **Nível B** | **Nível A** |  |
| --- | --- | --- | --- | --- | --- |
| **Informação os usuários em relação às diretrizes clínicas...** | ...não é realizada. | ...acontece quando solicitada ou por meio de publicações do serviço. | ...é feita por meio de material educativo elaborado para cada diretriz clínica. | ...é feita por meio de material educativo elaborado para os usuários de cada diretriz clínica, descrevendo seu papel – direitos e deveres - no cuidado à sua condição. | |
| **Pontuação** | 0 1 2 | 3 4 5 | 6 7 8 | 9 10 11 | |
| **Sistema de informação/**  **registro clínico…** | …não inclui resultados de avaliação do paciente ou metas de autocuidado apoiado. | …inclui resultados de avaliação dos usuários, como estado funcional e disposição de envolvimento no autocuidado apoiado, mas não inclui metas terapêuticas. | …inclui resultados de avaliação dos usuários, como estado funcional e disposição de envolvimento no autocuidado apoiado, além de metas terapêuticas. | … inclui resultados de avaliação dos usuários, como estado funcional e disposição de envolvimento no autocuidado apoiado, metas terapêuticas, além de alertas aos profissionais e aos usuários referente ao monitoramento da sua condição crônica e reavaliação periódica das metas terapêuticas. | |
| **Pontuação** | 0 1 2 | 3 4 5 | 6 7 8 | 9 10 11 | |
| **Programas comunitários...** | …não fornecem feedback para a instituição de saúde sobre o progresso dos usuários em suas atividades. | …fornecem feedback esporadicamente para a instituição de saúde sobre o progresso dos usuários em suas atividades. | … fornecem feedback regularmente para a instituição de saúde sobre o progresso dos usuários em suas atividades, por meio de instrumentos formais, por exemplo, relatórios na internet. | … fornecem feedback regularmente para a instituição de saúde sobre o progresso dos usuários em suas atividades, por meio de instrumentos formais, que são utilizados para modificar os programas de acordo com as necessidades dos mesmos. | |
| **Pontuação** | 0 1 2 | 3 4 5 | 6 7 8 | 9 10 11 | |
| **Plano organizacional para atenção às PVHA…** | …não utiliza uma programação local. | …usa dados dos sistema de informação clínica para planejar a atenção, mas com enfoque na programação da oferta. | …usa dados do sistema de informação clínica para planejar pró-ativamente o cuidado, com base populacional e estratificação de risco, derivados de parâmetros contidos nas diretrizes clínicas, incluindo autocuidado e parcerias com a comunidade. | …usa sistematicamente dados dos sistemas de informações para planejar pró-ativamente, com base populacional e estratificação de risco, derivados de parâmetros contidos nas diretrizes clínicas, incluindo autocuidado e parcerias com a comunidade e o desenvolvimento de um contrato de gestão com os profissionais de saúde, com base em desempenho e incluem a avaliação contínua do plano para determinar os avanços durante toda sua implementação. | |
| **Pontuação** | 0 1 2 | 3 4 5 | 6 7 8 | 9 10 11 | |
| **Monitoramento das metas dos planos de cuidado...** | …não é realizado. | …realizado esporadicamente, apenas para usuários interessados. | …é realizado por um profissional responsável ao usuário. | … é realizado por meio da designação de um profissional responsável ao usuário e usa o prontuário clínico e os alertas para coordenar a atenção entre o usuário e os membros da equipe. | |
| **Pontuação** | 0 1 2 | 3 4 5 | 6 7 8 | 9 10 11 | |
| **Diretrizes clínicas para as PVHA...** | ...não são compartilhadas com os usuários. | ...são fornecidas aos usuários que demonstram interesse na sua condição e no autocuidado apoiado. | ... são fornecidas a todas os usuários para ajuda-las a desenvolver o autocuidado efetivo ou nos programas educacionais de mudança de comportamento e para permitir que eles identifiquem quando devem procurar um profissional de saúde. | ...são compartilhadas pela equipe de saúde e pelos usuários para desenvolver o autocuidado efetivo ou nos programas educacionais de mudança de comportamento que levem em conta suas metas terapêuticas e a disposição para as mudanças. | |
| **Pontuação** | 0 1 2 | 3 4 5 | 6 7 8 | 9 10 11 |  |

Pontuação Total para Integração: _____________ Pontuação Média (Pontuação para Integração/6): _______________

**Resumo da Pontuação**

**(trazer as pontuações do final de cada sessão para esta página)**

1.Pontuação média para Organização da Atenção à Saúde _______

2.Pontuação média para Articulação com a Comunidade _______

3.Pontuação média para Autocuidado Apoiado _______

4.Pontuação média para Suporte à Decisão _______

5.Pontuação média para Desenho do Sistema de Prestação de Serviços _______

6.Pontuação média para Sistema de Informação Clínica _______

7.Pontuação média para Integração dos Componentes do Modelo de Atenção às Condições Crônicas _______

**Pontuação total (1+2+3+4+5+6+7) ______**_______

**Pontuação de capacidade institucional (1+2+3+4+5+6+7/7) ______**

**O que isto significa?**

O ACIC é organizado para que a pontuação mais alta (11) em qualquer item, seção ou avaliação final, indique um local com recursos e estrutura ótima para a atenção às condições crônicas. Por outro lado, a menor pontuação possível (0), corresponde a um local com recursos e estrutura muito limitados para a atenção às condições crônicas. A interpretação dos resultados é a seguinte:

**Entre “0” e “2” = capacidade limitada para a atenção às condições crônicas;**

**Entre “3” e “5” = capacidade básica para a atenção às condições crônicas;**

**Entre “6” e “8” = razoável capacidade para a atenção às condições crônicas;**

**Entre “9” e “11” = capacidade ótima para a atenção às condições crônicas.**

É comum que algumas equipes iniciem um processo de mudança com média abaixo de “5” em algumas (ou todas) as áreas do ACIC. Afinal, se todos ofertassem uma atenção excelente às condições crônicas, não seria necessário este processo ou outros programas para a melhoria da qualidade. Também é comum que equipes acreditem prover uma atenção às condições crônicas melhor do que realmente acontece. Com o desenvolvimento do processo, começam a familiarizar-se com o que um sistema de atenção efetivo envolve. Nesse caso, a pontuação ACIC pode até diminuir em vez de melhorar; mas isso é resultado do melhor entendimento do que deve envolver um bom sistema de saúde. Com o tempo, à medida que sua compreensão sobre atenção integral aumenta e a equipe continua a implementar mudanças efetivas, observará a melhoria de sua pontuação.
